# Treating Type 2 Diabetes Mellitus with Traditional Chinese and Indian Medicinal Herbs

**DOI:** 10.1155/2013/343594

**Published:** 2013-05-07

**Authors:** Zhijun Wang, Jeffrey Wang, Patrick Chan

**Affiliations:** ^1^Center for Advancement of Drug Research and Evaluation, College of Pharmacy, Western University of Health Sciences, 309 E. Second Street, Pomona, CA 91766, USA; ^2^Department of Pharmaceutical Sciences, College of Pharmacy, Western University of Health Sciences, 309 E. Second Street, Pomona, CA 91766, USA; ^3^Department of Pharmacy Practice and Administration, College of Pharmacy, Western University of Health Sciences, 309 E. Second Street, Pomona, CA 91766, USA

## Abstract

Type II diabetes mellitus (T2DM) is a fast-growing epidemic affecting people globally. Furthermore, multiple complications and comorbidities are associated with T2DM. Lifestyle modifications along with pharmacotherapy and patient education are the mainstay of therapy for patients afflicted with T2DM. Western medications are frequently associated with severe adverse drug reactions and high costs of treatment. Herbal medications have long been used in the treatment and prevention of T2DM in both traditional Chinese medicine (TCM) and traditional Indian medicine (TIM). This review examines *in vivo*, *in vitro*, and clinical evidence supporting the use of various herbs used in TCM and TIM. The problems, challenges, and opportunities for the incorporation of herbal frequently used in TCM and TIM into Western therapy are presented and discussed.

## 1. Introduction

Type 2 diabetes mellitus (T2DM) is a chronic illness due to endocrine dysfunction. Uncontrolled, diabetes is associated with various acute and chronic comorbidities. T2DM is a rapidly growing health concern in both developed and developing nations. T2DM accounts for over 90% of cases globally [1, 2]. According to the World Health Organization (WHO), in 2011, approximately 364 million people globally suffer from diabetes (DM), with projections that DM-related deaths will double from 2005 to 2030 [3]. In 2004, 3.4 million people died directly from the consequences of high blood glucose. The prevalence of DM worldwide was calculated as 2.8% in 2000. This is expected to increase to 4.4% by 2030 [4]. The growing concern is the epidemic growth in obesity and increase in the elderly population, which will continue to increase the prevalence of DM. Another study, using data from 91 countries, estimates that the prevalence can be as high as 7.7% (439 million adults) by 2030 [2]. Other estimates include a 70% increase in DM in developing countries and 20% increase in developed nations.

In the United States, T2DM is quickly becoming an epidemic. The Center for Disease Control (CDC) estimates that in the United States alone, 25.8 million Americans, or 8.3% of the population, suffer from DM, with 7 millions currently undiagnosed [5]. DM is higher, 26.9%, in the elderly (65 years or older). But it is also rapidly becoming a disease observed in younger patients with almost 2 millions over the age of 20 being newly diagnosed with DM in 2010. More alarmingly, 35% of adults over the age of 20 and 50% of elderly had prediabetes. This equates to 79 million people in the US. DM is the primary cause of renal failure, non-traumatic lower-limb amputations, and newly diagnosed retinopathy. DM is the 7th leading cause of mortality of Americans. 

### 1.1. Current Pharmacological Agents in the Treatment of T2DM

T2DM is a chronic disease that affects millions of people globally and is associated with multiple comorbidities and complications. DM education, prevention, and care are complex and should be designed to be patient specific. Physicians, nurses (and nurse practitioners), pharmacists, and dieticians are often recruited as a balanced health-care team in managing a patient's diabetes. The American Diabetes Association (ADA) promotes diabetes self-management education, a process in which the patient is equipped with the knowledge and skills to provide self-care, manage crisis (severe hyperglycemia and hypoglycemia), and make lifestyle changes [6, 7]. 

Primary non-pharmacological interventions include appropriate diet and exercise. Diet should be balanced and aimed to reduce weight. At least thirty minutes of moderate to intense exercise can improve T2DM and weight management. Intense lifestyle modifications (LSMs) are the mainstay of all treatment modalities and should be encouraged in both populations who are at risk for developing diabetes and patients who are suffering from diabetes. For patients requiring pharmacological interventions of T2DM, metformin, a biguanide, is first-line treatment for most patients who are unable to achieve their glycemic goals with LSM. Used for years, metformin increases glucose uptake by the skeletal muscles [8], inhibits hepatic gluconeogenesis [9], and increases insulin sensitivity [10] (summarized in Table 1). Not only is metformin the first-line recommendation for the treatment of T2DM, but there is also evidence of metformin being a useful agent in preventing T2DM in high-risk populations [11]. 

Sulfonylureas are a commonly used second-line class of antidiabetic drugs which increases insulin secretion by binding to K_ATP_ (potassium) channels of the *β*-islet cells in the pancreas [12]. Second-generation sulfonylureas are largely used due to their potency, fewer drug interactions, and less severe adverse reactions [6]. Insulin, which has long been considered last-line therapy in the treatment of T2DM and is the primary treatment of Type 1 Diabetes Mellitus (T1DM, insulin-dependent DM), is now a viable addition to metformin as a second-line agent in lieu of sulfonylureas [6, 7]. Insulin is effective in reducing blood glucose and HbA_1C_. Insulin regimens are patient-specific and can involve various combinations. 

Other less validated classes of medications that can be added to metformin include the thiazolidinediones (TZDs) and GLP-1 agonists (glucagon-like peptide-1). TZDs (pioglitazone, rosiglitazone) act by modulating peroxisome proliferator-activated receptor *γ* (PPAR*γ*), a nuclear receptor involved in the regulation of glucose and lipid metabolism. Activation of PPAR*γ* leads to increased insulin sensitivity primarily in adipose tissue but has also shown to have an effect on skeletal muscle and liver [13]. In the United States, TZDs carry a black box warning of increased cardiovascular events due to a trial that demonstrated an increased risk of myocardial events with rosiglitazone [14]. GLP-agonists (exenatide, liraglutide) are peptides derived from naturally occurring incretin hormones produced in the small intestines after meals [15]. It binds to GLP-1 receptors in the pancreas to stimulate insulin secretion and suppress glucagon secretion. The meglitinides class (repaglinide, nateglinide) has a similar mechanism to sulfonylureas [16] but binds to a different site from sulfonylureas on the K_ATP_ channels of the *β*-islet cells in the pancreas, also stimulating insulin release. There is a reduced risk of hypoglycemia with the meglitinides. *α*-Glucosidase inhibitors (acarbose, miglitol) work primarily in the gut by inhibiting *α*-glucosidase enzymes on the intestinal brush border. *α*-Glucosidase is a key enzyme for breaking down carbohydrates such as starch, dextrin, and disaccharides for absorption [17]. *α*-Glucosidase inhibitors may also stimulate GLP-1 secretion. Dipeptidyl peptidase-4 (DPP-4) is a protease enzyme responsible for the inactivation of hormones GLP-1 and GIP (gastric inhibitory peptide) [18]. Inhibition of DPP-4 by inhibitors (sitagliptin, saxagliptin, linagliptin) increases endogenous levels of GLP-1. Endogenous amylin and its analogues (pramlintide) bind to amylin receptors in the brain [19]. Endogenous amylin is secreted along with insulin from pancreatic *β*-islet cells. Pramlintide delays gastric emptying, reducing postprandial glucose levels [20]. 

### 1.2. Pharmacological Prevention of T2DM

The prevention of T2DM in patients primarily focuses on education, diet, and exercise. While the use of pharmacological approaches for prevention is not routinely practiced, the ADA recommends that health-care practitioners consider the use of metformin in patients who are at high risk for developing diabetes. In the Diabetes Prevention Program (DPP) trial, metformin 850 mg twice daily was given to female patients considered at risk for developing DM [11]. One group was administered metformin, another group underwent intensive LSM, the last group was given placebo medication. After a four-year study, the incidence of DM was decreased by 58%  (*P* < 0.001) with the LSM group and 31%  (*P* < 0.001) with the metformin-treated population when compared to placebo. As such, metformin is the only current medication that has been advocated to be used in the prevention of diabetes in high-risk populations such as those with a history of gestational diabetes, morbidly obese, and those with progressive hyperglycemia [6, 21].

The Troglitazone in Prevention of Diabetes (TRIPOD) study demonstrates preservation of pancreatic *β*-islet cell function [22]. TZD (troglitazone) was administered in high-risk Hispanic women as identified with the development of gestational diabetes within the previous four years. In women receiving 400 mg troglitazone for 30 months, the cumulative incidence of diabetes was reduced significantly in treated women (5.4%) compared to placebo (12.1%; *P* < 0.01). Troglitazone was discontinued in the USA in 1998 due to potential liver damage associated with the drug.

Over 1300 patients with impaired glucose tolerance in a multi-center study were selected for the STOP-NIDDM trial and given either acarbose three times daily or placebo [23]. After treatment for an average of 3.3 years, 17% of the patients in the acarbose-treated group developed diabetes compared to 26% in the placebo group (*P* = 0.001). 

Native Asian Indians with impaired glucose tolerance (IGT) enrolled in the Indian Diabetes Prevention Programme (IDPP-1) study received placebo, LSM, metformin, or LSM plus metformin [24]. Patients were followed for three years, and the cumulative 3-year incidences of diabetes were 39.3% with LSM (relative risk reduction [RRR] = 28.5%, *P* = 0.018), 40.5% with metformin (RRR = 26.5%, *P* = 0.029), and 39.5% with LSM plus metformin (RRR = 28.2%, *P* = 0.22). Results demonstrated that LSM or metformin alone can significantly lower the incidence of diabetes, but the combination of LSM and metformin did not display any added benefit. 

The IDPP-2 study recruited native Asian Indians with IGT and received LSM plus placebo or LSM plus pioglitazone. Followup three years later did not show improvements or reduction in the development of T2DM [25]. The cumulative risk was 29.8% in the pioglitazone group and 31.6% in the placebo group. 

In the DREAM trial (Diabetes Reduction Assessment with Ramipril and Rosiglitazone Medication), rosiglitazone was administered in hopes of preventing T2DM [26] in patients with IGT or impaired fasting glucose (IFG). Patients were followed for a median of 3 years. The incidence of DM in the rosiglitazone treatment group was 10.6% and 25% in the placebo group (*P* < 0.0001). The risk of T2DM or death was reduced by 60% in patients who have a high risk of developing T2DM. Heart failure, which is a concern of rosiglitazone, was 0.5% in the rosiglitazone arm compared to 0.1%  (*P* = 0.01) in the placebo arm. 

The NAVIGATOR (Nateglinide and Valsartan in Impaired Glucose Tolerance Outcomes Research) study group randomized patients with IGT to receive nateglinide or placebo with a median followup of 5 years [27]. The cumulative incidence of diabetes was nonsignificant in the nateglinide group (36%) compared to the placebo group (34%; *P* = 0.05). 

The effects of low-dose combination of metformin and rosiglitazone were examined in patients with IGT in the CANOE (Canadian Normoglycemia Outcomes Evaluation) trial [28]. The median followup was 3.9 years and demonstrated that this combination was effective in reducing the incidence of developing DM in the treatment group (14%) compared to the placebo group (39%; *P* < 0.0001), with a relative risk reduction of 66%. A significant reduction in insulin sensitivity in the placebo group (−1.24) compared to the treatment group (−0.39; *P* = 0.0006) was also observed.

Orlistat, a gastrointestinal lipase inhibitor used in the treatment of obesity, was used in the XENDOS (Xenical in the Prevention of Diabetes in Obese Subjects) trial [29]. Patients were recruited on the basis of BMI (body mass index) >30 kg/m^2^, which is classified as obese. Approximately 21% of the patients exhibited IGT in both the orlistat treatment group and the placebo group. The results of the four-year study showed the cumulative incidence of diabetes to be 6.2% in the orlistat-treatment group and 9.0% in the placebo group (37.3% risk reduction; *P* = 0.0032). 

### 1.3. Traditional Chinese Medicine (TCM) and Traditional Indian Medicine (TIM) for Treatment and Prevention of DM

Although there are currently a number of effective Western T2DM medications available for treatment, management of T2DM using medications with fewer side effects at lower costs is still a big challenge. These medications frequently have side effects, such as weight gain, bone loss, and increased risk of cardiovascular events [30]. These side effects could become more prevalent due to continuous use. Furthermore, treatment is very costly as well, since T2DM is a chronic disease and long-term medications are necessary. Herbal medications can be a good alternative to replace or at least supplement to Western medications [31–34]. The Indian and Chinese cultures have had several thousand years of history and experience in the prevention and treatment of T2DM with herbal medicine. As later discussed, several herbal medications have been proven to be clinically effective. Because herbal medicines are usually derived from natural plants, they are considered to be relatively safe and have fewer side effects compared to the conventional drugs. 

Herbal medications treating T2DM can target multiple mechanisms including enhancement of insulin sensitivity, stimulation of insulin secretion, or reduction of carbohydrate absorption [31]. Unlike Western medicine which usually contains a single active ingredient aiming for a specific mechanism, herbal concoctions may contain various active ingredients targeting multiple mechanisms. Herbal medicine is based on the holistic theory, which puts an emphasis on the integrated body. Western drugs are typically more potent than herbal medicine in lowering blood glucose levels. However, herbal supplements have shown to be able to treat diabetic complications [35]. Thus herbal medicine can also be used as supplementation or in combination with the Western medicine to improve better therapeutic outcomes. 

**Figure 1:**
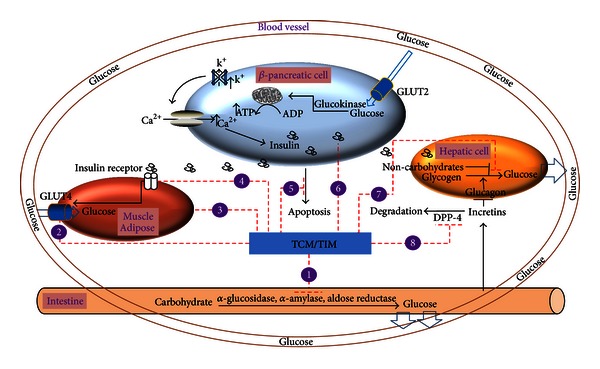
Mechanisms of antidiabetic effect of TCM and TIM herbs. (1) Reduced carbohydrate absorption, such as inhibition of *α*-glucosidase, *α*-amylase, and aldose reductase, (2) increased glucose uptake in muscle and adipose tissues, (3) activation of PPAR, (4) increased insulin sensitivity/upregulation of receptor expression, (5) exertion of antioxidant effects and decreasing *β*-cell apoptosis, (6) stimulation of *β*-cell insulin secretion, (7) inhibition of hepatic gluconeogenesis/glycogenolysis, and (8) prevention of endogenous incretins from degradation/suppression of glucagon. The numbered mechanisms of actions correspond to Table 1.

In the Chinese and Indian cultures, traditional medicine has long been the foundation in the treatment and prevention of many diseases. Approximately 800 plants have been identified in the treatment or prevention of T2DM. Many formulations are present as a single herbal extract or in a complex formula. Over 400 extracts have shown to be effective *in vitro* or *in vivo* [32]. The pharmacological mechanisms of the herbs can be classified as (1) decreasing carbohydrate absorption, (2) improving insulin sensitivity, (3) increasing peripheral glucose uptake, (4) stimulating insulin secretion, (5) potentiating endogenous incretins, (6) exerting antioxidant effects and decreasing cell apoptosis, and (7) increasing the glycogenesis or inhibiting hepatic glycogenolysis (Figure 1) [31, 32, 36]. Since many formulations contain multiple extracts and compounds, each herbal preparation may contain multiple mechanisms. In the following sections, we summarize the Chinese and English literature and list the most effective TCM and TIM herbal preparations under these identifiable mechanisms. Most of the studies have been conducted using *in vitro* systems and diabetic animals. However, there has been an increase in randomized placebo-controlled clinical trials testing the effectiveness of various TCM and TIM in both healthy and T2DM patients (Table 2). 

## 2. Commonly Used TCM/TIM for T2DM

### 2.1. Herbs in Both TCM and TIM

#### 2.1.1. *Gymnema sylvestre* Schult (syn. Periploca sylvestris Retz)

*Gymnema sylvestre *Schult, belonging to genus Gymnema and family of Apocynaceae, grows in the tropical forests of southern and central India, southern China, Vietnam, Australia, and African countries. The leaves of *G. sylvestre* have been used for treatment of diabetes, hypercholesterolemia, joint pain, and snake bites in India and China [37, 38]. The leaf extract of the *G. sylvestre* has also been marketed as herbal supplements for diabetic patients [39]. The major chemical components are gymnemic acids I-VII, triterpenoid saponins (gymnemosides A-F and gymnemoside W1-2), conduritol A, and dihydroxy gymnemic triacetate. 

The major bioactive constituents are gymnemic acids, a group of oleanane-type triterpenoid saponins including gymnemic acids I-VII, gymnema saponins and their derivatives such as deacylgymnemic acid (DAGA) which is the 3-O-glucuronide of gymnemagenin (3,16,21,22,23,28-hexahydroxy-olean-12-ene) [38]. 

In alloxan-induced diabetic mice, body weight as well as pancreas and liver weight were increased by oral administration of the leaf or callus extract of *G. sylvestre* at a dose of 200 mg/kg [39]. The effect of the extracts was similar to 4 unit/kg of insulin. Hepatic glycogen levels were also increased (2.15 to 2.47 mg/g versus 1.35 mg/g for the extracts and control, respectively), which in turn could stimulate the secretion of insulin. In streptozotocin (STZ)-induced DM rats, the hexane, acetone, and methanol extracts decreased plasma glucose levels. The acetone extract was found to be most potent. Oral administration of 600 mg/kg of the acetone extract for 45 days decreased the glucose level from 443 to 114 mg/L. Dihydroxy gymnemic triacetate was identified to be the major active component; at 5–20 mg/kg, it showed significant effects on lowering blood glucose level by increasing plasma insulin levels. *G. sylvestre* extracts were shown to be able to regenerate pancreatic *β* cells and increase circulating insulin level by stimulating its secretion [40].

In one small clinical study, the fasting blood glucose (FBG) and HbA_1C_ levels were improved in T2DM patients after receiving 200 mg of ethanolic extract of *G. sylvestre* either daily or their usual treatment for 18 to 20 months [41]. In a second clinical trial, the subjects showed reduced polyphagia, fatigue, blood glucose (fasting and postprandial), and HbA_1C_ in comparison to the control group following an oral dose of 500 mg of herbal extract for a period of 3 months [42]. In an uncontrolled trial involving 65 patients with T1DM and T2DM, the FBG and HbA_1C_ levels were decreased 11% and 0.6%, respectively, after oral dose of 800 mg daily of *G. sylvestre* extract [41]. 

#### 2.1.2. *Momordica charantia*

*Momordica charantia*, a tendril-bearing vine belonging to the Cucurbitaceae family (also known as bitter melon/gourd, karela, or balsam pear), is a popular plant used for the treatment of diabetes in China, South America, India, the Caribbean, and East Africa [32, 43].

In *M. charantia* seeds, the major components have been identified to be eleostearic acid and stearic acid, which account for approximately 45% of total weight. Several glycosides, such as charantin and vicine, were isolated from the *M. charantia* stem and fruit. Other components include polypeptide-p, lipids, triterpenoids, and alkaloids [43].

The methanol extract of *M. charantia* exhibited hypoglycemic effects in diabetic male ddY mice at a dose of 400 mg/kg. *M. charantia* can also suppress glucose tolerance and postprandial hyperglycaemia in rats [43] by inhibiting the absorption of carbohydrates from the gastrointestinal tract. 

Leung et al. reviewed clinical trials examining the hypoglycemic effects with *M. charantia *in T2DM patients. However, contradictory clinical outcomes were observed among these trials, probably due to poor methodological design without baseline characterizations along with non-standardized extraction method [43]. Nevertheless, the *M. Charantia* juice from the fresh fruit showed glucose-lowering effects in T2DM patients [44, 45], but not the extract from the dried fruit [46]. 

The extract of *M. charantia* using ethyl acetate was able to activate peroxisome proliferator-activated receptors (PPAR*α* and *γ*) and upregulate the expression of the acyl CoA oxidase gene in H4IIEC3 hepatoma cells. The suppression of peroxidation and apoptosis resulted in improvements in *β*-cell function and enhanced insulin excretion. In adipocytes, the momordicosides from *M. charantia* stimulated glucose transporter-4 (GLUT4) translocation to the cell membrane and increased the activity of adenosine monophosphate-activated protein kinase (AMPK), which could enhance glucose uptake from the blood. Animal studies showed that the extract could also enhance insulin sensitivity and lipolysis. In STZ rats, gluconeogenesis was inhibited by *M. charantia* via downregulation of hepatic glucose-6-phosphatase (G6P) and fructose-1,6-bisphosphatase activities [47]. 

#### 2.1.3. *Morus alba* L

The mulberry tree (*Morus alba* L.) grows widely in Asian countries, and various substituents of its leaves, *Folium mori*, have been applied clinically in TCM [48] as hypoglycemic, hypotensive, and diuretic agents. *Folium mori* have been traditionally used to treat hyperglycemia. The main bioactive components are flavonoids, alkaloids (1-deoxynojirimycin), and polysaccharides [33]. 

In T2DM mice (high sucrose-fed KK-Ay mice), *Folium mori* extract reduced insulin resistance following 8-week treatment. Both FBG levels and urinary glucose levels were significantly lowered in mice fed with a diet supplemented with Folium mori extract in a dose-dependent manner [49]. In Goto-Kakizaki rats, a spontaneous nonobese animal model for T2DM, the *Folium mori* extract demonstrated reduced postprandial blood glucose levels [50]. In human subjects, it showed that a food-grade mulberry powder enriched 1-deoxynojirimycin suppressed postprandial blood glucose serge [50, 51].

*In vitro* cell studies showed that in adipocytes, *Folium mori* extract increased glucose uptake and thus enhanced the translocation of GLUT-4 with concentrations ranging from 5 to 45 mcg/mL [52, 53]. In db/db mice, the extract ameliorated adipocytokines in white adipose tissue possibly due to the inhibition of oxidative stress [52]. One of the alkaloids, 1-deoxynorimycin, is also a potent inhibitor of *α*-glucosidase [54]. 

#### 2.1.4. *Trigonella foenum-graecum* L

The fenugreek is an annual plant in the family Fabaceae. The fenugreek seed was a traditional remedy used by ancient Egyptians and spread to Asian countries such as China and India [55]. Fenugreek seeds are a rich source of the polysaccharide galactomannan and also contain saponins such as diosgenin, yamogenin, gitogenin, tigogenin, and neotigogens. Other active constituents include mucilage, volatile oils, and alkaloids [56, 57].

The hypoglycemic effects of *T. foenum-graecum* in rats were firstly reported in 1974 [58]. Soon afterwards, the amino acid 2S,3R,4S, 4-hydroxyisoleucine, purified from fenugreek seeds, showed insulinotropic effects which increased peripheral glucose uptake *in vitro* [59–61]. The activities of hepatic enzymes hexokinase, glucokinase, G6P, and fructose-1,6-bisphosphatase were reduced in DM rats [62, 63]. Plasma glucose levels decreased after receiving the *T. foenum-graecum* extract in both non-DM patients and DM patients [64, 65]. Insulin levels were significantly higher in the fenugreek treatment group in comparison to the placebo treatment [66]. A meta-analysis of *T. foenum-graecum* showed that the herb may reduce HbA_1C_ by 1.13%  (*P* = 0.03) [67].

### 2.2. Other Herbs in TCM

#### 2.2.1. *Radix rehmanniae*

*Radix rehmanniae* is the root of *Rehmannia glutinosa *Libosch, under the family of Scrophulariaceae or Gesneriaceae. It has been widely used for treatment of diseases relating to blood, immune, endocrine, nervous, and cardiovascular systems.

The bioactive components of *Radix rehmanniae* include catalpol, rehmannioside A, B, C, and D, phenethyl alcohol derivatives such as leucosceptoside A and purpureaside C, monocyclic sesquiterpenes as well as their glycosides [68].

*Radix Rehmanniae* showed hypoglycemic activity in normal and STZ-induced DM mice. In Chinese medicine, it is usually prepared in combination with other herbs such as *Radix ginseng*, *Radix scutellariae* [69], and *Radix astragali* [70]. These combinations stimulated insulin secretion and *β*-cell proliferation through insulin receptor substrate 2 induction. It also showed improvements in diabetic foot ulcer healing in rats through the processes of tissue regeneration, angiogenesis, and inflammation control [70]. The postulated mechanisms of action are stimulation of insulin secretion, regulation of glucose metabolism in DM rats, and reduction of hepatic glycogen content of non-DM mice [31, 71]. 

#### 2.2.2. *Stephania tetrandra* Moore

*Stephania tetrandra* Moore is an herbaceous perennial vine of the Menispermaceae family, which is a fundamental herb used in TCM for the reduction of swelling and also providing an analgesic effect. The root of *S. tetrandra* has demonstrated to have anti-inflammatory, anti-allergic and hypotensive effects in experimental animal studies [72]. 

The major components are alkaloids, including tetrandrine, fangchinoline, bisbenzylisoquinoline, protoberberine, morphinane, and phenanthrene [73]. At 0.3–3 mg/kg, fangchinoline significantly decreased blood glucose and increased blood insulin in STZ-mice by potentiating insulin release [31]. In another study, formononetin, one of the active components in Radix astragali, potentiated the effect of *S. tetrandra* on lowering the blood glucose level and increasing the blood insulin level, although no direct anti-hyperglycemic effect of formononetin was observed [72]. The postulated antidiabetic mechanism of *S. tetrandra* extract is the stimulation of insulin release in pancreatic *β*-cells [72, 74]. 

#### 2.2.3. *Rhizoma coptidis*

*Rhizoma coptidis *is the rhizome of *Coptis chinensis *Franch that belongs to the Ranunculaceae family, recorded as *Coptidis Rhizoma* (CR) in the Chinese Pharmacopeia with the Chinese name of Huang Lian. It has been widely used to clear heat, dry dampness, and eliminate toxins from the body. It is also a commonly used herb in various formulas against intestinal infections, diarrhea, inflammation, hypertension, and hypoglycemia. 

The most well-known components of *Rhizoma coptidis* are isoquinoline alkaloid and berberine [75] which has variety of biological activities such as tumor reduction, anti-microbial, anti-Alzheimer's disease, anti-hyperglycemic, anti-inflammatory, and anti-malarial [76]. The berberine compounds of *Rhizoma coptidis* have been studied for its anti-hyperglycemic effects. The other alkaloids include palmatine, jateorrhizine, epiberberine, and coptisine.

Both the extract and pure berberine significantly decreased blood glucose and serum cholesterol levels in high fat diet-fed mice at the dose of 200 mg/kg by gavage. In alloxan-induced diabetic mice, berberine showed an anti-hyperglycemic effect and also blunted blood glucose increase induced by intraperitoneal glucose or adrenaline administration in normal mice. The activity of berberine was similar to sulfonylureas or biguanides [31, 77]. 

The anti-hyperglycemic effects of berberine could be due to the improvement of insulin sensitivity by activating the AMPK pathway or inducing insulin receptor expression. Berberine could also improve fatty acid oxidation via activation of AMPK and acetyl-CoA carboxylase. Furthermore, six quaternary protoberberine-type alkaloids of berberine inhibited aldose reductase activity *in vitro* with an IC_50_ less than 200 *μ*M [77, 78]. However, no evidence from *in vivo* studies is available to verify this mechanism.

#### 2.2.4. *Radix astragali*

*Radix astragali*, Chinese name of Huang Qi, is the dried root of perennial herbs *Astragalus membranaceus *(Fisch.) Bunge and *Astragalus mongholicus *(Fabaceae) Bunge of the Leguminosae family and grows in northern China. The major active compounds in *Radix astragali* are isoflavones and isoflavonoids (formononetin, calycosin, and ononin), saponins (astragaloside IV, astragaloside II, astragaloside I and acetylastragaloside), and astragalus polysaccharides [79]. 

*Radix astragali* possesses a broad spectrum of effects such as immunostimulation, hepatoprotection, diuresis, analgesia, expectorant, and sedation. In traditional Chinese medicinal theory, the herb is capable of consolidating the exterior of the body and can alleviate heat in the muscles by ascending positive qi [70, 80].

After treating DM Sprague-Dawley rats with *Radix astragali* decoction (500 mg/kg IP daily) for two months, improvements in insulin sensitivity and attenuation of fatty liver development were observed. However, blood glucose levels, *β*-cell function, and glucose tolerance were not substantially improved. *Radix astragali* polysaccharides reduced hyperglycemia and led to indirect preservation of *β*-cell function and mass via immunomodulatory effects in T1DM mice. In addition, its polysaccharides restored glucose homeostasis in T2DM mice/rats by increasing insulin sensitization. Formononetin, calycosin and ononin might exert a synergistic hypoglycemic effect with fangchinoline in STZ-diabetic mice, most likely by increasing insulin release [70, 80].

#### 2.2.5. *Eriobotrya japonica* Lindl

The loquat *Eriobotrya japonica* Lindl., a fruit tree in the family Rosaceae, is indigenous to central and south China. The dried leaves of *E. japonica*, also called *Folium eriobotryae*, have been used for treatment of chronic bronchitis, cough, and diabetes. The active compounds in *E. japonica* are identified to be triterpenes, sesquiterpenes, flavonoids, megastigmane glycosides and polyphenolic compounds including ursolic acid, oleanolic acid, cinchonain Ib, procyanidin B-2, chlorogenic acid, and epicatechin [81–83]. 

In an *in vitro* study using insulin receptor substrate-1 cells, the aqueous extract and the cinchonain Ib (one of the components in *E. japonica*) enhanced insulin secretion in a dose-dependent manner [84]. *In vivo* studies showed that the aqueous extract of *E. japonica* could transiently reduce blood glucose levels [84]. The 70% ethanol extract exerted a significant hypoglycemic effect on alloxan-diabetic mice following oral doses of 15, 30, and 60 g/kg (crude drug). The total sesquiterpenes were found to significantly lower blood glucose levels in both normal and alloxan-diabetic mice [85]. Shih et al. found that the extract, with major components of tormentic acid, maslinic acid, corosolic acid, oleanolic acid, and ursolic acid, could ameliorate high fat induced hyperglycemia, hyperleptinemia, hyperinsulinemia and hypertriglyceridemia [86]. Another study found that the co-fermentation of *Folium eriobotryae* and green tea leaf reduced the blood glucose level by 23.8% within 30 min in maltose-loaded SD rats at a dose of 50 mg/kg, although this effect was not observed in the sucrose- and glucose-loaded rats [87]. 

#### 2.2.6. *Ginkgo biloba*

The *Ginkgo biloba* tree, native to China, dates back to the prehistoric ages of 250–300 million years and is frequently called a “living fossil” [88]. Today, the biloba tree can live more than 1,000 years [89]. In the United States, *G. biloba* is one of the most frequently used over-the-counter (OTC) herbal supplements [90]. Extract from its leaves contains ginkgo flavonoid glycosides, terpene lactones, and ginkgolic acids. Many human clinical trials have examined possible ginkgo uses in cerebrovascular disease, tinnitus, sexual dysfunction, intermittent claudication, migraine prophylaxis, and alleviating symptoms of the common cold [15, 91–94]. However, the most common use of ginkgo is the prevention and treatment of Alzheimer's disease and dementia [95–100]. 

The administration of the ginkgo extract, EGb 761, in rats with DM increased glucose uptake into hepatic and muscle tissues [101] and decreased atherogenesis, a common comorbidity of DM [102]. *In vitro* assays determined the possible anti-diabetic effect of ginkgo to be through the inhibition of *α*-glucosidase and amylase activities [103]. Clinical investigations of the anti-diabetic properties of ginkgo in humans have produced mixed results. Healthy human subjects showed no reduction in blood glucose levels with an accompanying significant increase in plasma insulin levels [104]. The randomized double-blinded clinical study involving non-DM, pre-T2DM, and T2DM patients showed that ginkgo did not increase insulin sensitivity nor reduced blood glucose levels [105, 106]. However, in T2DM patients, ingestion of *G. biloba* extract showed increased clearance of insulin, resulting in a reduction plasma insulin levels and elevated blood glucose. Gingko may improve endothelial function in T2DM patients with early stages of nephropathy, but without affecting blood glucose levels [107]. While popular for its many possible indications, gingko appears to have limited anti-diabetic properties to warrant its use in diabetes. 

#### 2.2.7. *Radix ginseng*

*Radix ginseng* is native in the northern hemisphere, most notably, in eastern Asia (northern China, Korea, and eastern Siberia) and northern America. Subsequently, ginseng is often named from its origin—Asian ginseng, American ginseng, Chinese ginseng, to name a few. More than 700 compounds have been identified in ginseng, with the most active components being identified as the ginsenosides (Rb1, Re, Rd), polysaccharides, peptides, and polyacetylenic alcohols [108]. The geographical origin of ginseng, in combination with the extraction and processing method, produces variable anti-diabetic results [32, 108, 109]. 

The anti-diabetic activity of *Ridix ginseng* has been explored in both animal and human studies. Hypoglycemic activity is greater in lipophilic extracts than aqueous extracts. In DM rats, Korean ginseng (0.1–1.0 g/mL) stimulated the release of insulin from isolated pancreatic islets. American ginseng (100 mg/kg) produced lowered levels of serum glucose and HbA_1C_ in DM rats [110]. Vuksan and colleagues have conducted a number of human clinical trials demonstrating that ginseng reduced postprandial blood glucose, fasting blood glucose, and HbA_1C_ levels [111–115]. Similar results were presented at American Diabetes Association Annual Meeting in 2003 [116]. 

Pharmacologically, ginseng has antioxidant properties. It also reduces *β*-cell apoptosis by upregulating adipocytic PPAR-*γ* protein expression [117]. Ginseng impairs glucose absorption by decreasing glucosidase activity [118]. It may also increase insulin sensitivity in peripheral tissues [32]. One of the active components, ginsenoside Rb1, can enhance glucose transport by inducing the differentiation of adipocytes via upregulating the expression of PPAR-*γ* and C/EBP-*α* [119]. In addition, ginsenoside Rb1 can increase GLUT-4 activity leading to increased uptake of glucose from blood by adipocytes [120].

#### 2.2.8. *Fructus schisandrae*

*Fructus schisandrae* (also known as “five-flavor berry” in China), the fruit of a deciduous woody vine native to forests of northern China, is traditionally used as a tonic or sedative agent. It has been used in TCM to astringe the lungs and nourish the kidneys. It was reported that *Fructus schisandrae* can enhance hepatic glycogen accumulation and decrease hepatic triglycerides. It has also been used in various TCM formulas, such as the modified Ok-Chun-San and modified Huang-Lian-Jie-Du-Tang, to treat diabetes [121]. The major chemical components include lignans such as schizandrins (schizandrin A) and gomisins (gomisin A, J, N, and angeloylgomisin H), and polysaccharides [72], 

*In vitro*, gomisin J, gomisin N and schizandrin A increased basal glucose uptake in HepG2 cells [122]. Several other schizandrins were found to be able to prevent *β*-cell apoptosis and decrease insulin resistance [123]. In an *in vitro* study using 3T3-L1 adipocytes, several fractions of ethanol extract showed the stimulation effect of PPAR-*γ*. Among these fractions, FS-60, a subfraction from the 70% ethanol extract, was identified to be most potent with the major components of schizandrin A, gomisin A, and angeloylgomisin H. In an *in vivo* study using pancreatectomized DM rats, FS-60 lowered serum glucose levels during the OGTT similar to the level of the fasting stage. During hyperglycemic clamp, FS-60 increased the first phase insulin secretion in diabetic animals [121]. 

The major mechanisms are hypothesized to be the stimulation of insulin secretion and increased insulin sensitivity by ameliorating insulin resistance via increased PPAR-*γ* activity. *Fructus schisandrae* can also improve glucose homeostasis in DM mice by inhibiting aldose reductase [121]. 

#### 2.2.9. *Pueraria lobata* (Gegen)

Gegen is the dried root of *Pueraria lobata* (Willd.) Ohwi, a semiwoody, perennial and leguminous vine native to South east Asia, and also known as yegen, kudzu root, and kudzu vine root [124, 125]. For more than 2000 years, gegen has been used as an herbal medicine for the treatment of fever, acute dysentery, diarrhea, DM, and cardiovascular diseases [126, 127]. Over seventy compounds have been identified in gegen, with isoflavonoids (puerarin) and triterpenoids being the major constituents.

*In vitro* studies showed that puerarin contained in *P. lobata* can enhance the glucose uptake in a dose-dependent manner performed in high glucose-treated preadipocytes [128]. Puerarin also promoted insulin-induced preadipocyte differentiation and upregulated mRNA expression of PPAR*γ*, which can regulate glucose homeostasis, adipocyte differentiation, and lipid metabolism [129]. In China, a clinical trial was conducted in DM patients using the Gegen Qin Lian decoction which showed a dose-dependent effect on reducing HbA_1C_ and FBG [130]. Possible mechanisms of action from *in vitro* and *in vivo* studies include *α*-glucosidase inhibition, increased expression and activity of PPAR-*γ*, upregulation of GLUT-4 mRNA, increased plasma endorphins, and preservation of pancreatic islets [131–133].

#### 2.2.10. *Cornus officinalis* Sieb. et Zucc

*Cornus officinalis *Zucc., native to China, Japan, and Korea, is a common herbal medicine of the family of Cornaceae. Fructus corni is the dried ripe sarcocarp of *C. officinalis* Sieb. et Zucc. Cornaceae, which has been widely prescribed as a tonic agent in Chinese medicinal formula and possess activities of improving the function of the liver and kidney [134]. The major active components are iridoid glycosides, morroniside, loganin, mevaloside, loganic acid, ursolic acid and oleanolic acid, 5-hydroxymethyl-2-furfural, and 7-O-galloyl-D-sedoheptulose [135].

The ethanol extract of Fructus corni induced the expression of GLUT-4 by stimulating the proliferation of pancreatic islets, resulting in increased insulin secretion [136]. One of the active components, ursolic acid, was found to be an inhibitor of protein tyrosine phosphatase (PTP) 1B, which sensitizes the effects of insulin [137]. The Fructus corni extract decreased blood sugar in STZ mice and reduced renal oxidative stress and glycation products in STZ-induced diabetic rats. The underlying mechanisms include the inhibition of glucosidase, reduction of gene expression for hepatic gluconeogenesis, protection of *β*-cells against toxic challenges, and enhancement of insulin secretion.

### 2.3. Other Herbs in TIM

#### 2.3.1. *Barringtonia racemosa*

*Barringtonia racemosa* is an evergreen mangrove tree that grows in Bangladesh, Sri Lanka, and the west coast of India, with the bark and leaves used for snake bites, rat poisoning, boils, and gastric ulcers. The extracts from different parts have various biological activities including anti-cancer, analgesic, anti-DM, anti-bacterial, and anti-fungal activities. Its seeds are aromatic and useful in colic and ophthalmic disorders [138].

Several diterpenoids and triterpenoids have been identified in *B. racemosa* extract and a pentacyclic triterpenoid, bartogenic acid, is the major active component [138, 139]. The hexane, ethanol and methanol extracts as well as the pure compound of bartogenic acid inhibited intestinal *α*-glucosidase activity at concentrations ranging from 0.02–0.2 *μ*g/mL in an *in vitro* enzymatic study. In an* in vivo* rat study, the methanol extract was found to suppress the rise of blood glucose level after receiving maltose [140]. 

#### 2.3.2. *Syzygium cumini* (L.) Skeels

*Syzygium cumini *(L.) Skeels, frequently referred to as Skeels, is a tropical tree native to India, China, and Indonesia. Skeels is also known as *Eugenia jambolana*, Jamun, Jambu, Black Plum, or Black Berry and has been frequently used to treat DM in India [140] and Brazil [141]. Studies using DM rats showed reductions in blood glucose, post prandial glucose, cholesterol, and free fatty acid [142, 143]. Pharmacologically, the extracts of *S. cumini* have shown *α*-glucosidase inhibitory activities [144, 145]. Hepatic enzymatic activities of glucokinase and phosphofructokinase, hepatic enzymes which play a role in glucose metabolism, were significantly reduced in DM animals [145, 146]. Adenosine deaminase activity was inhibited in Skeels-treated erythrocytes procured from both DM and non-DM patients. However, clinical trials have not produced favorable results. Two double-blind, randomized trials involving non-DM and DM patients did not support the use of Skeels in DM [147, 148]. In patients with DM consumed tea prepared from *S. cumini* leaves, FBG levels were not reduced significantly. 

#### 2.3.3. *Tinospora cordifolia*

*Tinospora cordifolia*, also called Guduchi of the Menispermaceae family, is a succulent climbing shrub, indigenous to the tropical areas of India, Myanmar, and Sri Lanka. The aqueous stem extract is used for curing gastrointestinal pain [149]. *T. cordifolia* also has anti-spasmodic, anti-pyretic, anti-allergic, anti-inflammatory, immunmodulatory, and anti-leprosy activities [150]. The bioactive ingredients are alkaloids (palmatine, jatrorrhizine and magnoflorine), diterpenoid lactones, glycosides, steroids, sesquiterpenoid, phenolics, aliphatic compounds, and polysaccharides. 

The anti-DM activity of *T. cordifolia *has been investigated in DM mice. The aqueous and alcoholic extract of the plant can improve glucose tolerance in DM rats. Grover and coworkers found that *T. cordifolia* ameliorated diabetic neuropathy at a dose of 400 mg/kg [151]. The 70% ethanol extract significantly decreased blood glucose levels and attenuated the rate of blood glucose elevation after 2 g/kg glucose loading following an oral dose of 100 or 200 mg/kg of *T. cordifolia *for 14 days [152]. The extract was also found to be able to prevent diabetic retinopathy in STZ diabetic rats at a dose of 250 mg/kg [153].

The anti-DM effect is related to the amelioration of oxidative stress by reducing the production of thiobarbituric acid-reactive substances. The extract can increase the expression of thioredoxin and glutaredoxin. *T. cordifolia* extract can also adjust alter carbohydrate metabolism and reduce gluconeogenesis via inhibiting G6P and fructose 1,6-diphosphatase [154]. Other possible mechanisms examined are the enhancement of the insulin release and inhibition of *α*-glucosidase [155].

#### 2.3.4. *Ocimum basilicum*

*Ocimum basilicum*, with the common name of basil, or sweet basil, is a culinary herb of the family Lamiaceae (mints), which is sometimes known as Saint Joseph's Wort. Basil was originally from India and widely used in Southern Asian. 

Basil is a potent anti-septic and preservative agent and also demonstrates slight sedative effects, regulation of digestion, and diuresis. Clinically, it has been used to treat headache, cough, upper respiratory tract infection, and kidney dysfunction. Laboratory studies have found that basil also has activities in lowering blood sugar, stimulating nervous system, and protection from radiation [156, 157]. 

The major components of basil consist of apigenin, linalool, and ursolic acid, which previously demonstrated anti-viral activity [158]. Basil improved lipid metabolism in hypercholesterolemic rats [159]. In an *in vitro* cell line study using human macrophages, the ethanol extract of basil reduced cholesterol synthesis [160]. The aqueous extract of *O. basilicum* can inhibit rat intestinal sucrase, maltase, and porcine pancreatic *α*-amylase activities which may have positive effect for treatment of DM [161]. In a clinical trial in DM patients in India, the basil leaf extract decreased the fasting blood glucose by 21.0 mg/dL, and postprandial blood glucose fell by 15.8 mg/dL. The results suggest that *O. basilicum* may be used as a dietary therapy in mild to moderate T2DM [162].

#### 2.3.5. *Berberis aristata*

*Berberis aristata* (also known as Zarshik, Daruharidra) of the family Berberidaceae is an Ayurvedic herb which has been used since ancient times in South Asia as an herbal tonic agent to improve hepatic and cardiac functions [163, 164].

The main constituents of the root have been identified as berberine, berbamine and palmatine [165]. The extract of *B. aristata* (root) has a strong potential to regulate glucose homeostasis by decreasing gluconeogenesis and oxidative stress. In DM rats, the extract increased the glucokinase and G6P dehydrogenase activities but decreased G6P activity [165]. In patients with sub-optimal glycemic control, HbA_1C_, basal insulin, insulin resistance, total and low-density lipoprotein cholesterol, and triglycerides were significantly reduced after 90-day treatment with combination of *B. aristata* extract and *Silybum marianum* extract [166]. 

## 3. Problems, Challenges, and Opportunities

There are two entirely different approaches in the future research on TCM/TIM. The “sharp shooter” approach is to select a particular plant with a specific activity or a biological target. By using bioactivity-guided isolation and structural elucidation, one can discover a new chemical entity for a specific disease target. Many Western drugs, such as chemotherapeutic agent paclitaxel, were discovered this way, while some others such as metformin were developed upon further structural modification. Obviously, this is a validated approach for drug discovery and development for the treatment of human diseases. On the other hand, instead of targeting a specific receptor or mechanism, one can select proper combinations of herbs or ingredients, and optimize the outcome of treatment by different combinations and dose regimens. This approach is termed the “shotgun” approach. While it is not necessary to identify the exact active ingredient(s), it is still necessary to have good quality control of the preparations to ensure reproducibility. Certain chemical markers in the preparations can be selected as markers to standardize the raw materials and processing procedures. Once a reproducible preparation is obtained and its biological activity is established, it can be used to treat certain given disease in the general patient population. Because the extract contains multiple components which may interact with multiple disease targets, it might be advantageous to a single chemical entity, especially in the area of disease prevention.

However, pharmaceutical scientists are facing unique challenges in developing herbal products as anti-DM agents.

1. *Patentability*. Because herbal medicines derive from natural plants, their active components cannot be patented as novel materials. It is also difficult to patent their usage since much information is already in the public domain. But it is possible to patent a unique combination and/or the extraction process.
2. *Product standardization*. This should be achieved via proper control on raw material, extract process and final formulation. Without effective quality control, consistency of the herbal product may be compromised. Improved methods for quality control of herbal products, such as bioactivity-guided pharmacokinetic methods and genomic fingerprinting techniques are promising.
3. *Placebo-controlled, randomized clinical trials*. TCM/TIM physician's philosophy to individualize formula for different patients has significantly hindered the systematic scientific investigation according to Western medicine standards. In comparison to their Western counterparts, the anti-DM efficacies of TCM/TIM herbs have not been well studied using randomized, double-blinded clinical trials, although many animal studies have been carried out. However, the implementation of placebo-controlled, randomized clinical trials is a prerequisite for the evidence-based practice of using TCM/TIM preparations for DM prevention.
4. *Toxicity and herb-drug interaction*. TCM herbs are generally thought to be relatively safe and with milder side effects. However, their activities are usually not as potent as Western medications. Thus high doses (sometimes as high as 10 g per day) are usually required to achieve optimal therapeutic efficacy. In addition, the toxicity of herbal products cannot be ignored. Most common complaints of herbal supplements ingestion are gastrointestinal related, including stomach upset, diarrhea, constipation, nausea, and vomiting. In addition, more serious adverse effects may also occur. For example, ginseng abuse syndrome is a result of chronic ingestion of excessive amounts of ginseng. This is characterized by hypertension and CNS stimulation, insomnia, and nervousness. There is also an increased awareness of herb-drug interaction in pharmacokinetics and pharmacodynamics. When used in combination with established anti-DM medications, herbal supplementation may predispose patients at an increased risk of hypoglycemia. For example, *Ginkgo biloba* extract interacts with selective serotonin reuptake inhibitors used in the treatment of depression, resulting in the serotonin syndrome, and with thiazide diuretics resulting in decreased efficacy.

It is encouraging to note that two drugs based on plant extract have been approved by the FDA for the treatment of human diseases. Veregen (Polyphenon E) Ointment is the first prescription botanical drug approved by FDA in 2006. It is an extract of green tea as a prescription drug for the topical (external) treatment of genital warts caused by the human papilloma virus (HPV). More recently, FDA's approval of crofelemer (Fulyzaq) signals the first time an orally administered botanical has received drug approval from the Administration. Crofelemer derived from the latex of the South American sangre de drago tree (dragon's blood, Croton lechleri) is the first drug to be approved in the United States to treat HIV-associated diarrhea. With experience gained through the developmental and regulatory processes comes high hope that many TCM/TIM-based anti-DM products will be available for the general population.

## 4. Conclusions

It is evident that many TCM/TIM herbs possess anti-DM activities by interacting with various proven drug targets where Western drugs interact. Because of their empirically known oral efficacy and safety profiles, nutritional supplement status, multiple components for multiple drug targets, low cost, and easy access, TCM/TIM herbs such as ginseng, mulberry, and *Radix coptidis* are excellent candidates for long-term use for the prevention and treatment of T2DM. During the development stage, product standardization, quality control and assurance, placebo-controlled and randomized clinical trials are essential components that need to be perfected in order to translate their potential into a reality that millions of people could benefit upon.

## Figures and Tables

**Table 1:** Comparison of various mechanisms of action of Western medications with TCM and TIM herbs. Numbers in parenthesis corresponds with mechanisms of action depicted in Figure 1.

	Inhibition of carbohydrate absorption (1)	Increased peripheral glucose uptake (2)	Activation of PPAR (3)	Increased insulin receptor expression (4)	Increased insulin receptor sensitivity (4)	Decreased peroxidation or apoptosis of *β*-cells (5)	Stimulation of insulin secretion (6)	Decreased gluconeogenesis/glycogenolysis (7)	Suppression of glucagon (8)	Delayed gastric emptying
Western medications										
Biguanides		∗			∗			∗		
Sulfonylureas							∗			
Thiazolidinediones			∗							
GLP-agonists							∗		∗	∗
Meglitinides							∗			
*α*-glucosidase inhibitors	∗									
DPP-4 inhibitors							∗		∗	∗
Amylin										∗
Herbs										
*G. sylvestre *							∗			
*M. charantia *	∗	∗	∗			∗		∗		
*F. Mori *	∗	∗								
*T. foenum-graecum *		∗					∗	∗		
*Ridix Rehmanniae *							∗	∗		
*S. tetrandra *							∗			
*Rhizoma coptidis *	∗			∗	∗					
*Radix astragali *					∗		∗			
*E. japonica *							∗			
*G. biloba *	∗	∗								
*Radix ginseng *		∗	∗			∗	∗			
*Fructus schisandrae *	∗		∗		∗	∗	∗			
*P. lobata *	∗	∗	∗			∗				
*C. officinalis *	∗	∗			∗	∗		∗		
*B. racemosa *	∗									
*S. cumini *	∗							∗		
*T. cordifolia *	∗						∗	∗		
*O. basilicum *	∗									
*B. aristata *								∗		

**Table 2:** Recently completed and current clinical trials of herbs.

Herb	Trial name	Status	Sponsors	Clinical trial no.
*Gymnema sylvestre* Schult.	Double Blind Randomized Trial to Compare Gurmar (Gymnema sylvestre) with Metformin in Type 2 Diabetes	Status currently unknown	(1) Postgraduate Institute of Medical Education and Research (2) Indian Council of Medical Research (3) International Clinical Epidemiology Network (INCLEN) TRUST	NCT00396851

*Momordica charantia*	The Effect of Metamin 3D on the Lipid and Glucose in Subjects with Metabolic Syndrome	Completed 2009	Taichung Veterans General Hospital, Taiwan	NCT01120873

*Folium mori* (1-deoxynojirimycin extract)	Effect of Mulberry Leaf Extract on Blood Glucose	Completed 2011	(1) Ewha Womans University (2) Bundang CHA Medical Center (3) Ministry of Knowledge Economy, Korea	NCT01385865

*Trigonella foenum-graecum *L.	Effect of Fenugreek on Blood Sugar and Insulin in Diabetic Humans	Completed 2008	(1) Pennington Biomedical Research Center (2) Louisiana State University Health Sciences Center in New Orleans	NCT00597350

*Rhizoma coptidis *	Trial of Different Dosages' Ge Gen Qin Lian Decoction in the Treatment of Type 2 Diabetes	Currently recruiting patients	Guang'anmen Hospital of China Academy of Chinese Medical Sciences	NCT01219803

*Rhizoma coptidis* (berberine extract)	Efficacy and Safety of Berberine in the Treatment of Diabetes with Dyslipidemia	Completed 2006	Shanghai Jiao Tong University School of Medicine, China	NCT00462046
	Therapeutic Effects of Berberine in Patients with Type 2 Diabetes	Completed 2004	(1) Shanghai Jiao Tong University School of Medicine (2) National Institutes of Health (NIH)	NCT00425009

*Ginkgo biloba *	Ginkgo Biloba Extract and the Insulin Resistance Syndrome	Completed 2005	National Center for Complementary and Alternative Medicine (NCCAM)	NCT00032474

*Radix ginseng* Mey (ginsenosides extract)	A Clinical Trial of Ginseng in Diabetes	Completed 2008	Washington University School of Medicine	NCT00781534

Data retrieved from the U.S. National Institutes of Health (http://clinicaltrials.gov/).
